# An in-depth analysis of pharmaceutical regulation in the Republic of Moldova

**DOI:** 10.1186/2052-3211-7-4

**Published:** 2014-05-01

**Authors:** Alessandra Ferrario, Nina Sautenkova, Zinaida Bezverhni, Rita Seicas, Jarno Habicht, Panos Kanavos, Vladimir Safta

**Affiliations:** 1 Department of Social Policy, London School of Economics and Political Science, Houghton Street, London WC2A 2AE, UK; 2 LSE Health, London School of Economics and Political Science, Houghton Street, London WC2A 2AE, UK; 3 WHO Regional Office for Europe, World Health Organization, UN City, 51 Marmorvej, Copenhagen 2100, Denmark; 4 Department of Social Pharmacy "Vasile Procopisin", State Medical and Pharmaceutical University "Nicolae Testemitanu", 22 Testemitanu Street, Chisinau 2025, Republic of Moldova; 5 Center for Health Policies and Studies, 99/1, V. Alecsandrii Street, Chisinau 2012, Republic of Moldova; 6 WHO Country Office in Republic of Moldova, World Health Organization, 29 Sfatul Tarii Street, Chisinau 2012, Republic of Moldova

**Keywords:** Access, Regulation, Medicines, Republic of Moldova, Former Soviet Union

## Abstract

**Objective:**

Regulation of the pharmaceutical system is a crucial, yet often neglected, component in ensuring access to safe and effective medicines. The aim of this study was to provide an in-depth analysis of the existing pharmaceutical regulation, including recent changes, in the Republic of Moldova.

**Methods:**

Data from field work conducted by the World Health Organization (WHO) together with a review of policy documents and quantitative secondary data analysis was used to achieve this aim.

**Results:**

This analysis identified several ways in which pharmaceutical regulation affects availability of quality medicines in the Republic of Moldova. These include lack of full implementation bioequivalence requirements for generics registration, incomplete implementation of good manufacturing practices and no implementation of good distribution practices, use of quality control instead of quality assurance as a method to ensure quality of medicines, frequent change of power within the Medicines and Medical Devices Agency (MMDA) leading to lack of long-term strategy and plans, conflict of interest between the different functions of the MMDA, the lack of sufficient funding for the MMDA to conduct its activities and to invest in continuous training of its staff (particularly inspectors) and very weak post-marketing control. Notably, several improvements have been recently introduced, including a roadmap for change for the MMDA, the introduction of good manufacturing practices and the drafting of a quality manual for the Agency.

**Conclusion:**

Based on these findings the authors propose a set of priority actions to address existing gaps and draw lessons learned from other countries.

## Introduction

The Republic of Moldova is a lower-middle-income country in eastern Europe with a population of 3.5 million (1st of January 2013, data do not include the districts on the left side of the river Dniester and the municipality of Bender) [1] and a gross domestic product of US$ (current) 2,038 per capita in 2012 [2]. The economy is heavily based on remittances from Moldovan citizens working abroad. The country was part of the former Soviet Union, from which it obtained independence in 1991. Since independence, Moldova, including its health sector, has undergone profound social, political and economic transformations. While some reforms were introduced in the hospital sector, much remains to be done [3] and the most important changes so far were compelled by the introduction of a mandatory health insurance system in 2004 [4]. These included strengthening of primary health care and ensuring access to a limited number of medicines in privatised pharmacies and selected national programmes (e.g. insulin, rare diseases, tuberculosis (TB), HIV/AIDS, immunisation)[5-7].

Regulation of the pharmaceutical system is a crucial, yet often neglected, component in ensuring access to safe and effective medicines. While medicines financing, selection and procurement are essential, an effective, transparent regulatory system which manages conflict of interest (COI) and has a well-functioning post-marketing system (including product a recall system and pharmacovigilance) is crucial to prevent entry of substandard products. The latter pose a threat to patients’ health and cause wastage of resources spent on ineffective and/or unsafe products. The importance of regulatory control in ensuring equitable access to essential medicines is also included in the World Health Organization (WHO) framework for collective action [8].

Despite the importance of regulatory functions in ensuring access to essential medicines, few studies are available which explore pharmaceutical regulatory issues in countries of the former Soviet Union. These include a 1996 review of the transparency levels across regulatory systems worldwide which looked at Estonia and Latvia [9], a 1998–9 review of regulatory systems worldwide including Estonia [10], a study analysing fees charged by regulatory authorities which included Latvia [11], a more recent study (2008) on pharmacovigilance activities including Belarus, the Republic of Moldova, the Russian Federation and Ukraine [12] and two studies on the quality of information provided on websites of drug regulatory authorities which included Estonia, Latvia and Lithuania [13,14].

The objectives of this study are twofold. First, to provide an in-depth analysis of the existing pharmaceutical regulation, including recent changes, in the Republic of Moldova by reviewing gaps and progress in the main pharmaceutical system functions. Second, using the case of the Republic of Moldova, this paper aims to highlight the importance of pharmaceutical regulation in ensuring access to quality medicines. As issues of transparency and gaps in regulatory pharmaceutical functions are common in most low- and middle-income countries, this paper is expected to stimulate the debate in the area and encourage similar analysis in other countries.

## Methods

This analysis is based on two in-country missions (field work) conducted by the WHO and complemented by a desk-review of official reports, policies and regulations, as well as analysis of data on the number of registered medicines vs. available medicines obtained from the national laboratory of quality control and the Medicines and Medical Devices Agency (MMDA). The secondary data analysis was conducted in 2013 and enabled an update on findings from the missions. In 2011, the MMDA was only responsible for pharmaceuticals and was called Medicines Agency. In this paper, we will use the new name of the agency (MMDA) but limit our analysis to issues related to pharmaceuticals.

The first mission focused on procurement and supply and was conducted by two pharmaceutical experts between the 26th of June and the 1^st^ of July 2011. The aim of this field study was to analyse the centralised procurement process undertaken by the MMDA, through interviews with seven management and staff members of the MMDA. In addition, a number of structured interviews with major stakeholders were undertaken. These included representatives (number of interviewees in brackets) from the Ministry of Health (3), private importers and distributors (2), a public importer (1), the Oncological Institute (1), the central district hospital in the rayon of Calarasi (rural, 1), a primary health care unit in the rayon of Calarasi (2), the Republican clinic hospital (urban, 2), a private pharmacy (1), a manufacturer (1) and an academic with expertise in pharmaceutical policy in the Republic of Moldova (1).

The second mission was an extensive review of the entire regulatory system for pharmaceuticals and was conducted between the 19th and 23rd of September 2011 by three pharmaceutical experts (including a regulator from a European Union (EU) country). The review was performed using a WHO data collection tool for the assessment of regulatory systems [15,16]. Information was collected through interviews using the WHO data collection tool as a guide. Interviews were conducted with staff members from the main regulatory institutions in the Moldovan pharmaceutical sector and representatives of professional associations of pharmacists. National pharmaceutical legislation and websites of main regulatory bodies were also reviewed. The data obtained through interviews were used to validate and complement data obtained from secondary sources. By using the WHO data collection tool for the assessment of regulatory systems, the main gaps and progress identified in the different pharmaceutical functions were identified.

## Results

There is a lack of transparency in the regulation of medicines. This is characterised by low access to information on regulations and procedures for applicants and holders of marketing authorisation and lack of clear standard operating procedures (SOPs) for several regulatory procedures. Although various steps have been taken in recent years to address these gaps, the main challenges are the time needed to introduce the necessary legislative changes and low human resource capacity for their implementation. In fact, despite various guidelines having been drafted, many of them have not been approved yet; and for those approved, their implementation is often limited due to human resource constraints. In the next sections, we will highlight some of the main gaps and achievements across the various regulatory functions.

### Marketing authorisation (registration) of medicines

The main issue is the conflicting role of the MMDA which is tasked to register products and also to procure them. In a few cases, this has led to pressure from manufacturers to register products quickly. Further, the legal provision requiring the demonstration of generics’ bioequivalence with the brand product is not implemented. This is mainly due to the lack of assessors trained in reviewing biowaver^a^ applications for highly soluble substances and to insufficient attention from authorities to the problem. Good manufacturing practice (GMP) inspections are currently not a mandatory part of the registration process and there is insufficient funding and staff trained with the necessary training to conduct GMP inspections. Due to low salaries and general paucity of skilled professionals in the country, there is an insufficient number of assessors with relevant skills in the registration department and the national reference laboratory. In addition, the objectivity of the decisions taken by assessors is weakened due to lack of approved written procedures on how to assess registration applications.

Another issue is the lack of stable management or a long-term planning process within the MMDA. In the past two years, there have been seven different directors and despite the staff’s genuine commitment to raise standards and meet internationally accepted requirements, there is very little evidence of planning and targets at the MMDA or department level to achieve these goals. Further, there are no COI guidelines for registration activities, rendering the system vulnerable to vested interests. This can be particularly problematic given the frequent use of external experts. Moreover, the committee’s decisions are not publicly available.

**Figure 1:**
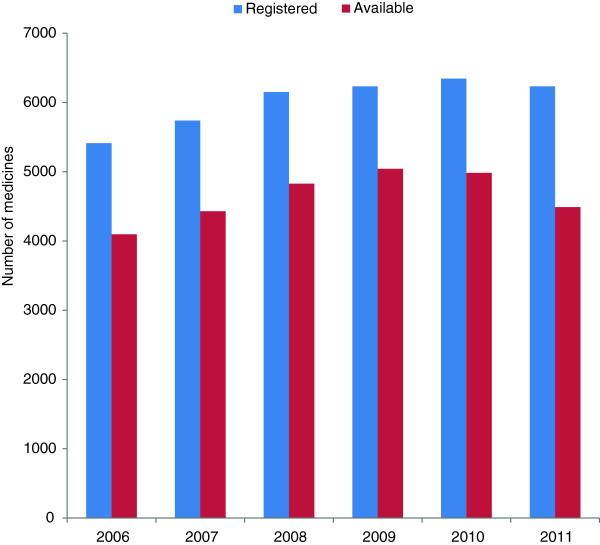
**Number of medicines registered by foreign manufacturers vs. available ones.** Source: Authors’ compilation based on data from the National Laboratory of Quality Control.

On average, about 70% to 80% of all registered medicines by foreign manufacturers were actually imported (Figure 1). However, analysis of the number of registered products for different international non-proprietary names (INNs) suggests that in many cases more products are registered than necessary. One of the most extreme examples is amlodipine 5 mg and 10 mg, for which, in 2012, there were 47 and 42 products registered by foreign manufacturers and 20 and 21 products imported, respectively. A less extreme example, yet still making the case, is metformin 500 mg and 850 mg, for which, in 2012, there were 16 and 15 products registered by foreign manufacturers and 5 and 5 products imported, respectively (Additional file 1: Table S1).

In most cases when a particular medicine was only registered by foreign manufacturers, even if not all strength were available, at least one strength per INN was imported. In a few cases, mostly cancer medicines, no product for any strength was available for a particular INN. These included bleomycin (not imported in 2012 and 2011), busulfan (not imported in 2012), cetuximab (not imported in 2012 and 2011), cytarabine (not imported in 2012), docetaxel (not imported in 2012), doxorubicin hydrochloride (not imported in 2012), everolimus (cancer indication not imported in 2012 and 2011), fludarabine (not imported in 2012 and 2011), imatinib (not imported in 2012 and 2011) and tamoxifen (not imported in 2012).

Unavailability of registered medicines is due to a number of reasons including unsuccessful pricing negotiations between manufacturer and the MMDA and disincentive on the part of distributors who may not have sufficient interest in importing certain medicines because of limited or absent demand. Low demand can be due to different reasons like irrational prescribing, heavy promotion of other medicines (outside the essential medicines list (EML)) and limited promotion of essential medicines. Finally, the financing of the MMDA is mainly based on registration fees which is likely to provide an incentive to register more products that actually necessary. Underfunding of the MMDA is a real challenge given that registration fees have not been revised in recent years (and thus does not take into account current expenses and inflation rate). The issue of low availability of registered medicines has been already discussed at Ministry of Health and some measures have been taken. Following the recent introduction of a new regulatory requirement in 2012, if a medicine is registered but it is not available on the market (whether it has never been launched or it stops being available) for three consecutive years, its registration is suspended [17].

#### Positive developments

Despite challenges remaining, a number of positive developments have taken place in the past two years. The most important positive development is the introduction of GMP guidelines, which, by December 2014, should be implemented by all local manufacturers [18]. In July 2012, the Ministry of Health approved a new regulation for medicines registration which includes a requirement to use the common technical document (CTD). This document contains all information on quality, safety and efficacy required by ICH^b^ regulatory authorities in a standard format accepted in all ICH member countries [19]. Since January 2013, all registration dossiers are organised and presented according to the CTD format. In addition, the MMDA is developing an e-application system for registration. This should help with tracking progress and compliance with timelines. Concerning registration fees, a draft document with a revised fee structure and additional fees for activities not previously included (e.g. fee for GMP inspection, fee for pharmacovigilance related activities, etc.) has been drafted and presented to Government for approval in August 2013 but has not yet been approved.

### Licensing of pharmaceutical enterprises

The Licensing Chamber, a public organ under the Ministry of Economy is currently responsible for licensing of pharmaceutical enterprises. This leads to fragmentation of responsibilities between the MMDA, the Licensing Chamber and the National Evaluation and Accreditation Office. A transfer of the licensing function to the MMDA was envisaged in the Medicines Agency Road Map for 2012–2014, which was approved by the Government in April 2012. However, a draft document to modify the law on pharmaceutical licensing activities was never approved due to lack of support from the other ministries. In addition to not have the licensing function under a specialised authority, as is the case in other countries, there are also no pharmacists within the licensing Chamber.

On the 1st of April 2011, the Parliament approved new rules for opening a pharmacy. The new requirements included a minimal distance between pharmacies and location to be based on demographic criteria. The law also required the Government to approve, within three months, a national plan to determine the location of new pharmacies based on these new criteria. The Ministry of Economy was opposed to this national plan and the idea of involving the Government, mainly arguing that regulating the opening of new enterprises is against free market principles. It thus provided negative feedback on the proposed plan and proposed to instead develop a new revision of the law to cancel all the new criteria. The Ministry of Health developed the new draft law which revoked the demographic criteria for pharmacy opening locations as well as the national plan. However, the final text currently under review by the Parliament will likely result in the removal of all three stipulations as requested by the Ministry of Economy. It is therefore still unclear whether any new criteria on opening new pharmacies will be introduced. Regardless, even if new criteria are approved, complexities in access would still remain and thus further considerations could be needed. For example, based on population criteria, the municipality of the capital Chisinau had too many pharmacies, calling for a moratorium on new openings. In 2012 there were 1,838 inhabitants per pharmacy in the capital rayon (district) of Chisinau vs. an average of 4,488 inhabitants per pharmacy in the other rayons. But in reality, there were certain villages near the capital (still part of the municipality of Chisinau) or in the recently-built ‘microrayons’ (‘microdistricts’) where the number of pharmacies per capita was too low. However, because the average number of pharmacies in the rayon was too high, it was not possible to open new pharmacies in those undersupplied areas.

### Inspections and market control

There are currently no written procedures or mechanisms in place to prevent regulatory capture between inspectors and the manufacturers or distributors that they inspect, rendering the system vulnerable to corrupt practices. While inspectors follow available regulations, these do not provide detailed description of the procedure to conduct inspection (SOPs). Another important gap is the lack of a quality management system for the inspectorate. Written criteria for the selection and recruitment of inspectors are available, requiring inspectors to have passed a specific training in inspection. Once recruited, inspectors are not regularly trained on key areas such GMP, good distribution practices (GDP) or good clinical practice (GCP). Further, there is no documented process on qualification and review to confirm the competencies of existing inspectors.

The legislation relating to MMDA inspectors involvement in pre-licensing activities is unclear and inspectors are not involved in any stages preceding the opening of a pharmacy. Further there is no clear legal provision designating the inspectors and their powers. As a result, inspectors do not have adequate powers and authority to carry out their tasks. Another issue relates to the fragmentation problems mentioned previously in that only the Licensing Chamber, and not the MMDA, can initiate the process to revoke a license.

Due to an insufficient number of competent staff and underfunding, the MMDA does not have resources to inspect manufacturers with the frequency stated in the law. Importing of low cost medicines from countries like India and China has increased in recent years, yet there is very limited capacity to inspect overseas suppliers and to raise questions about the quality of the products imported. This problem has now been addressed for reimbursed outpatient medicines (as explained in the next paragraphs in the positive developments subsection) but remains a challenge for inpatient medicines and non-reimbursed medicines.

The recall system for faulty products is not well described in the law and there are no SOPs. As a result, its implementation does not work well in practice; for example, suppliers who underperform are not blacklisted. Based on doctors’ and patients’ reports, there is evidence of the presence of low efficacy products in hospitals. This leads to overuse, increase of dosage or to patients having to purchase medicines, which they should receive for free at the hospital, in private pharmacies. These circumstances are supported by findings during one of the missions, when three different products with the same batch number from a local manufacturer were found in a hospital. There were also specific reports of low effectiveness in three different medicines: ceftazidime, an antibiotic imported from China which required two to three times the standard dose to elicit the response which is usually obtained with the standard dosage; methyl prednisolone and betamethasone, both locally produced corticosteroids (from a non-GMP compliant local manufacturer) which were reported to have no effect on patients.

#### Positive developments

The Republic of Moldova has already taken concrete steps to gradually move towards GMP compliance for imported as well as locally produced medicines. The first step was the development and approval of GMP guidelines in March 2013. With the release of the new reimbursement list in May 2013, medicines registered in the EU, the United States of America (USA), Canada, Australia, or Japan (and therefore compliant with GMP) were given preferred supplier status. This is expected to provide a strong incentive, particularly to local manufacturers, to improve their manufacturing practices according to internationally accepted GMP. Yet, in the case no product is registered from a GMP compliant manufacturer, products from manufacturer with national GMP certificates or, as a last resort, non-GMP compliant manufacturer need to be sourced. As a result of this, a number of medicines from non-GMP compliant manufacturer (particularly a local manufacturer which produced the two products discussed before) are still in the reimbursement list. For inpatient medicines, all products tendered should comply with GMP (no specification whether national or according to the WHO, European Medicines Agency (EMA) or Food and Drug Administration (FDA) criteria). The exception is psychotropic and TB medicines for which GMP certification according to either WHO, EMA or FDA is required. Further, by December 2014 all local manufacturers are expected to comply with GMP requirements. This will help to gradually remove existing double standards within the country as revealed during the procurement and supply mission, where an exporting facility was found to have voluntarily developed a quality assurance (QA) system because GMP compliance is required for product registration in the majority of the countries where the company exports its products. On the contrary, a domestically-oriented facility visited did not implement GMP (this was the local manufacturing facility which produced the two products mentioned previously, methyl prednisolone and betamethasone). These efforts were complemented with training organised for inspectors in GMP and GDP.

Improvements were also observed in the way inspections are conducted. The new law on state control of entrepreneur activity, implemented in March 2013, includes a provision for inspection of enterprises (which includes also pharmaceutical establishments). It defines the principles of control and the procedures to conduct controls. As of March 2013, the Inspectorate is publishing the schedule of visits to pharmacies and warehouses. Moreover, the old procedure (as per the previous legislation) to inspect the pharmaceutical facilities has been revised; now, to visit a pharmaceutical establishment the inspector has to inform the facility five days in advance of the planned visit.

Positive developments in access to narcotics for pain management have also occurred. Previously, the control over the supply chain of narcotic substances used to be too strong and fear of sanction was acting as a deterrent for pharmacists to stock narcotics. This situation was likely to cause problems for patients needing narcotic painkillers, particularly in rural areas where availability is low. In October 2012, the Ministry of Health released an order which introduced important changes in the regulation on dispensing narcotics medicines. Limits on the quantity of medicine prescribed were removed; doctors can now issue prescriptions for up to 30 days. Doctors can now also prescribe additional doses before the 30 day treatment period ends, in case the patient condition has changed.

### Medicines promotion

Control on medicines promotion is an important regulatory area in Moldova where doctors are known to be heavily influenced by pharmaceutical representatives through financial incentives. Due to very low salaries (the average salary of a primary health care doctor was Lei (Moldovan) 3,500 (US$ 260) in 2012) the latter can easily double physicians’ income making this practice very difficult to eradicate without first addressing the problem of low wages. Beyond financial issues, lack of training in rational prescribing and evidence-based medicine make physicians easy preys of the pharmaceutical industry. This is compounded by a lack of guidance from the public institutions in areas such as patient education and disease self-management which further strengthened industry’s role and influence. For example, doctors are required to run diabetes and hypertension schools but they do not have a standardised curriculum and are not provided with patient education materials to distribute so they rely on the pharmaceutical industry to provide patient education materials.

There is a law on medicines (# 1409 from 1997/12/17 in the chapter VI) providing general regulation on medicines’ information and advertising. The draft of regulation and executive order which sets out definitions, requirements, procedures of authorisations, prohibitions, monitoring and complaints procedure on advertising on medicines was presented at the beginning of 2012 to the Ministry of Health for approval and is currently still under discussion. The law on advertising (# 1227 from 22.06.1997) outlines the general principles for advertising, including provisions on adverting medicines and commodities. The provisions on medicine promotion and advertising include explicit mention of the different forms of promotion but post-marketing scientific studies, speakers’ fees and consultancies, promotion of exported medicines and restrictions and limits on gifts and gimmicks. In addition, the provisions do not foresee an enforcement mechanism on promotion and advertisement of medicines, stating the sanctions in cases of violation. In addition to legal gaps, there are also procedural ones. For example, while a formal complaints procedure to report unethical promotional practices exists, there is no evidence that this is used. Further, despite a standard check list for pre-approval and monitoring is available, it does not provide SOPs and there are gaps in the guidelines on COI for committee members or public officials involved in the control of medicine promotion activities.

A revision of the law on advertising was launched by a group of deputies in early 2012 with a proposal to prohibit advertising of medicines on television. The Government accepted the proposal but it was refused by the Parliament. As in other regulatory areas, a number of other regulations remain in draft form pending approval the Ministry of Health. These include the number of samples pharmaceutical representatives are allowed to provide, advertisement on the internet, enforcement rules and training of medial or sales representatives.

#### Positive developments

A draft of the quality manual of MA has been developed.

### Procurement

The main problem in this area is the COI between the MMDA’s regulatory and procurement functions but there are also various gaps in availability of guidelines, SOPs and monitoring systems. For example, there are no guidelines for staff on tendering processes and although a general law on COI is available, there are no specific guidelines for the procurement process. The regulation on quality control does include requirements for each shipment to be physically checked and samples taken, but there are no SOPs for routine inspection of consignments. There is a computerised management information system to report product problems during the procurement process but it does not include quality assurance information. Further, there is no centralised system to track the status of each order or to compare quantities purchased with orders. Suppliers’ performance is monitored at least annually but there is no system to track suppliers’ lead-time, shelf-life, packaging of products and pre- and post-qualification of suppliers. It is not straightforward to prevent underperforming suppliers to take part in tenders again. In one instance, a supplier who failed to supply the market at the agreed conditions participated again in the tender process the following year under a different corporate name.

While the Republic of Moldova has started using morbidity data and treatment guidelines information for forecasting purposes, procurement is still mainly based on previous consumption data which, as a method, has various limitations. To be reliable, this method requires a stable supply system with a relatively uninterrupted supply and a full supply pipeline [20]. It also means that is for any reasons some medicines were not procured in one year, they will not be considered in the next forecasting round.

National tendering is used to procure hospital medicines but there are often very few bidders and sometimes no bidder at all. In a few instances, when only one bidder was available in both rounds, the price in the second tendering round was higher because the bidder took advantage of their monopoly position, forcing the MMDA to accept the second price offer. One of the main reasons is certainly the limited advertising of tenders, only using local channels and in local language. A second possible reason goes back to the regulatory gaps identified previously which can act as a disincentive for manufacturers to launch their products due to non-transparent procedures and decision-making criteria. A third possible reason is the underdevelopment of the regulation ground to allow for the use of procurement methods other than centralised tendering. For specific products with limited market share, alternative methods such as price negotiation or single source procurement from international procurement agencies could be better suited. Finally, the small market size and low income levels are unlikely to make the market particularly attractive.

Although it is important from a transparency perspective to have public scrutiny by another body on procurement activities, the need for the Public Procurement Agency under the Ministry of Finance to approve each step in the procurement process and to sign all the contracts has caused delays to the tendering process in the past. This is true also for the very close, and often obstructive, involvement of the Anti-Corruption and Anti-Monopoly agencies at all stages of the tendering process. Another issue due to misplaced transparency is the current practice of sharing bids with bidders. This practice was introduced to increase transparency, but could prove deleterious as it may lead to collusion between manufacturers.

#### Positive developments

Stating in November 2012, new regulations are in place for use of additional public procurement methods including framework agreements, competitive dialogue, negotiation procedure and electronic tender. These were already mentioned in the law but government-approved implementation procedures were missing.

### Selection

There is no consistent procedure and criteria for development of the EML, leading to a copy and paste approach of the WHO standard EML without adjusting it to actual needs of the country. Further, there are no efforts in place to promote prescribing based on EML among physicians. There are several gaps regarding selection of committee members including the lack of a declaration of COI, no definition of the role and responsibilities of committee members and the absence of SOPs to guide the selection committee’s decision-making process.

#### Positive development

The EML is currently being updated to better reflect the epidemiological profile of the country. Further, the medicines reimbursement list is under increased scrutiny from a number of national and international partners. This could help increasing the level of accountability and transparency in the selection process and, hopefully, the extent to which such criteria are evidence-based and following rational selection principles.

### Distribution

The main gap is the lack of GDP which threatens the maintenance of medicines’ quality during the distribution process. Although the country is planning to introduce GDP guidelines, these have not yet been approved. Further, there are no SOPs for stock management at each level of the distribution system and it is unknown to what extent the requirements mentioned in the law are implemented at the warehouse level.

### Quality control

Issues related to the use of quality control rather than quality assurance measures have caused delays in availability of medicines because every imported batch had to be tested (which is not necessary and causes work overload for the laboratory). Further, SOPs for quality control are largely not available and a quality policy for the laboratory is missing. Another important gap is the lack of a strong and well-functioning post-marketing quality control system.

#### Positive development

The country has recently taken some very initial, yet promising, first steps in moving towards a risk-assessment and quality assurance system with the introduction of a new order on quality control of medicines. With the new order, medicines registered by companies from the EU, Australia, Japan, Canada or the USA are tested on a random basis (previously all first ten batches). For medicines registered by companies from a country other than EU, Australia, Japan, Canada, or USA but compliant with national GMP, all the first five batches are tested (previously all first ten batches). If the company passes all these first five tests, all following tests are done on random (but not risk assessment) basis. For manufacturers which are not compliant with GMP, all batches are tested, not only the first five. Transition to quality assurance should hopefully help avoid delays in the future.

### Import and exports

Currently, price is taken into consideration before granting an import authorisation. This practice should be discontinued as price is not an element which should influence the release of import permissions.

### Pharmacovigilance

Low implementation of drug monitoring poses a considerable risk for patients, as pre-marketing studies cover only a very small and highly selective proportion of all the patients who will use the drug in real life and are therefore not large enough to capture all possible side effects. Despite pharmacovigilance being part of the medical curriculum and the availability of reporting forms on the MMDA website, reporting is very low. This seems to be due to a lack of understanding of the importance of reporting, poor collaboration between medical institutions, a culture of fear of making contact with authorities, lack of pharmacovigilance responsibilities for manufacturers (though a new act is under development), lack, until recently, of SOPs on pharmacovigilance activities and unclear use of the data collected. Another issue is understaffing within the MMDA which is responsible for pharmacovigiliance.

#### Positive developments

After being stable for several years, between 2004 and 2011 the number of reports to the pharmacovigilance department at the MMDA has increased from 72 in 2011 to 180 in 2012 (of which 171 were reported to the international monitoring centre in Uppsala). While these levels are very low even for a country of the size of Moldova, this is still an encouraging signal. In 2012, 30% of all adverse drug reactions (ADR) reports were for TB medicines, 25% for antiretroviral drugs (ARV), 11% for antibiotics, 7% for medicines to induce labour and reduce post-partum haemorrhage, 4% contraceptives, 4% antihypertensives and the remaining percentage for other therapeutic groups [21]. While it is not possible to compare the increase in reporting by therapeutic group due to lack of disaggregated data for previous years, most of the ADR reports in 2012 were for TB and ARV drugs (55% combined), which is likely to be linked with the trainings organised by the Global Fund to fight AIDS, tuberculosis and malaria (GF) and WHO in these two therapeutic areas. Capacity building of health staff including management of side effects and reporting is part of the GF support to the country in the area of TB and ARV treatment. In addition to that, in 2011, WHO organised a training course on pharmacovigilance for ARV drugs in Kiev and participants (doctors involved in ARV treatment and two specialists from the MMDA) became enthusiastic and started sending reports. Although the increase in reporting is a positive result, more efforts are needed to improve reporting across all disease areas. Other positive developments include the recent adoption by the MMDA of the Vigi Flow system and the introduction of a fee for pharmacovigilance activities, which were previously funded from the general budget of the MMDA.

## Discussion

### Implications for access to quality medicines

Similar issues affecting the medicines regulatory system in the Republic of Moldova have been identified in other former Soviet Union countries. These include fragmentation of regulatory responsibilities across different agencies and unclear coordination mechanisms, lack of sustainable funding for the Medicines Agency, gaps in key regulatory documents (e.g. regulations, guidelines, SOPs) which lead to weak implementation of regulations and lack of transparency in decision-making, absence of COI policy and code of conduct for internal staff and external experts, no or partial implementation of GMP, GDP and GCP, insufficient number of trained staff and lack of continuous training, weak to absent market control function, large gaps in the quality management system in terms of both availability of key documents and implementation, insufficient staff and funding to conduct pharmacovigilance activities and lack of strategic planning for the Medicines Agency (unpublished observations)^c,d,e,f,g,h^.

This review of the Moldovan regulatory system for pharmaceuticals highlighted several gaps but also showed that progress has been made over time in a number of areas. It appears, however, that more effort has been paid to developing regulations and less in assuring the effectiveness of their implementation and their impact. That said, it is important to acknowledge some of the factors which limit the country’s ability to invest in effective implementation. These include the small population size, high levels of emigration (among both the highly skilled and low skilled labour force) and brain drain to the private sector within the country, which means that there is only a very small number of experts available (e.g. inspectors). Further, frequent changes of power, which lead to high staff turnover, undermine the strengthening of existing structures and systems. This is exacerbated by limited resources for public agencies to perform their duties, low salaries (which again contribute to high staff turnover but also make physicians more prone to industry’s pressures and incentives) and examples of nepotism within the ruling elite of public agencies.

Despite several positive steps forward in addressing regulatory gaps, the existing pharmaceutical legislation remains unstructured and not harmonised with EU requirements. In August 2013, the Ministry of Health approved a working group for revision of medicines legislation. A road map for 2012–2014 was also developed, which includes revision of existing regulations to meet EU requirements. The national drug policy has not been updated since 2002 and awareness among staff at the MMDA seemed to be low during the regulatory mission. Updating the policy is now one of the priorities of the collaborative agreement 2012–2013 between WHO Europe and the Republic of Moldova [22]. A draft of the pharmaceutical code exists but has yet to be finalised or implemented.

Finally, the absence of a well-functioning pharmacovigilance system and the presence of a weak post-market quality control system are jeopardising patient safety and quality of medicines in the Moldovan market. A survey of pharmacovigilance activities in 55 low- and middle- income countries showed that lack of budget, understaffing and lack of training were among the main challenges identified [12]. Low reporting was an issue in a number countries, with 15 countries having received less than 100 adverse drug reactions report and 30 countries less than 1000 reports in 2007 [12]. Experience with TB and ARV in the Republic of Moldova shows that it is possible to increase the number of ADR reports through training of health professionals. These efforts should be extended to all disease areas by providing training in pharmacovigilance to health staff (particularly doctors) and explaining, through concrete examples, the importance of pharmacovigilance and the positive impact of reporting so as to motivate doctors to engage in this activity. Reporting by patients should also be encouraged.

Gaps in regulation and lack of transparent procedures are some of the main reasons for constant interference of other organisations in the work of the MMDA. This often delays the MMDA in carrying out its regulatory functions and, together with other issues such as low salaries, is largely responsible for the very high turnover of the staff. Low levels of transparency in pharmaceutical regulation are an important issue in almost all newly independent states. Low levels of transparency in regulatory procedures provide opportunities for people with vested interests to influence the decision making process. To a large extent that also explains the permanent presence of law enforcement agencies like the Anti-Corruption and Anti-Monopoly agencies, which are also often used by politicians as private vehicles to put pressure on other agencies. Further, lack of transparency weakens the MMDA position, particularly in the eyes of foreign manufacturers, and is likely to act as a deterrent for the latter to do business in the country and participate in tenders.

As well free from political pressure, medicines regulation should also dispose of the necessary resources for its operation [23]. Yet, financial independency and sustainability of medicines regulatory authorities is an issue in a number of developing countries. Analysis of registration fees in 34 countries suggested that for new drugs in developing countries these fees could be raised to one to five time the gross national income and used a policy instrument to retain high qualified staff and reduce registration times without turning into a disincentive for the pharmaceutical industry [11]. Finally, it appears that the position of the Ministry of Health is not very strong in comparison to other ministries (e.g. the Ministry of Agriculture which is the main driver of the economy), as the direct and especially indirect economic impact of good health is not monitored and used for macro political decisions.

## Conclusions

This analysis identified several ways in which pharmaceutical regulation affects availability of quality medicines in the Republic of Moldova. These include lack of full implementation bioequivalence requirement for generics registration, incomplete implementation of GMP and no implementation of GDP guidelines, use of quality control instead of quality assurance as a method to ensure quality of medicines, frequent change of power within the MMDA leading to lack of long-term strategy and plans, COI between the different functions of the MMDA, the lack of sufficient funding for the MMDA to conduct its activities and to invest in continuous training of its staff (particularly inspectors) and very weak post-marketing control.

Based on the experience of the Republic of Moldova, the following lessons learned can be drawn for countries facing similar challenges. First, there is a need to address existing regulatory and developing SOPs where missing. This will allow for increased levels of transparency in the system, particularly regarding the objectivity of decision-making processes. Second, it is important to develop a strong quality management system based on quality assurance and implementing GMP, GDP and GCP to ensure the quality of the drugs reaching patients. Third, there is a need to focus not just on developing regulations but most importantly, to ensure that they are implemented. For this to happen, key regulatory agencies need to have sustainable funding and trained people at their disposal, have access to continuous training opportunities and be free from political pressures and finally enjoy stable management which will facilitate long term planning. Fourth, different procurement methods, depending on the drug to be procured, may be needed to ensure availability of essential medicines. Fifth, more efforts are needed to strengthen pharmacovigilance systems. These include financial and human resources but also training for doctors, nurses and patients on the importance of pharmacovigilance along with simplifying reporting requirements and making the necessary reporting forms widely available through different media. Sixth, this analysis showed that there are challenges affecting access to medicines which go beyond the pharmaceutical and health sector such as brain drain and the low level of salaries which affects the entire economy. Joint work with other Ministries is required to address such challenges.

### Endnotes

^a^The term biowaiver is applied to a regulatory drug approval process where the efficacy and safety part of the dossier (application) is approved based on evidence of equivalence other than through in vivo equivalence testing (http://apps.who.int/prequal/info_applicants/info_for_applicants_BE_implementation.htm).

^b^International Conference on Harmonisation of Technical Requirements for Registration of Pharmaceuticals for Human Use (ICH).

^c^Raudsepp K, Polishchuk O, Bolokhovets G. Review of the medicines regulatory system in Georgia. Tbilisi, Georgia: World Health Organization Regional Office for Europe, 2013.

^d^Azatyan S, Sautenkova N. Review of the Medicines Regulatory System of Kyrgyzstan. Bishkek, Kyrgyzstan: World Health Organization, 2008.

^e^Azatyan S, Sautenkova N, Polishchuk O. Assessment of the Medicines Regulatory System of Turkmenistan. Ashgabat, Turkmenistan: World Health Organization, 2009.

^f^Reggi V, Polishchuk O, Azatyan S. Review of the Medicines Regulatory System of the Republic of Armenia. Yerevan, Armenia: World Health Organization, 2007.

^g^Stará D. Review of the Medicines Regulatory System with focus on HIV/AIDS and TB medicines and related commodities in Ukraine. Kiev, Ukraine: WHO/EURO, Delegation of EU Commission in Ukraine and USAID2008.

^h^Stará D. Review of drug regulatory system in Republic of Belarus. Minsk, Republic of Belarus: World Health Organization Regional Office for Europe, 2009.

## Abbreviations

ARV: Antiretrovirals; COI: Conflict of interest; CTD: Common technical document; EML: Essential medicines list; EMA: European Medicines Agency; EU: European Union; FDA: Food and Drug Administration; GF: Global Fund to fight AIDS, tuberculosis and malaria GMP, good manufacturing practices; GDP: Good distributional practices; GCP: Good clinical practices; MMDA: Medicines and Medical Devices Agency; SOPs: Standard operating procedures; QA: Quality assurance; TB: Tuberculosis; WHO: World Health Organization.

## Competing of interests

The authors declare that they have no competing interests.

## Authors’ contributions

AF and PK had the initial idea for the paper and defined its scope. NS and VS collected primary and secondary data during the two country missions supported by JH; RS, ZB and VS collected secondary data from policy reports, registration and quality control data. AF, NS, RS, ZB analysed the data. AF wrote the first draft of the manuscript, redrafted subsequent versions based on all the co-authors comments and finalised the manuscript. All the authors critically reviewed the manuscript and provided inputs on subsequent versions based on their expertise. All authors read and approved the final manuscript.

## Supplementary Material

Additional file 1: Table S1 A comparison of medicines registered by foreign manufacturers vs. available ones by therapeutic indications (2012). Source: Authors’ compilation based on data from http://nomenclator.amed.md/ (for registration status) and the MMDA (for import status). Notes: Due to lack of data on availability of locally produced medicines, we had to limit the analysis of registered vs. available medicines to imported products. Click here for file

## References

[B1] Website of the National Bureau of Statistic of the Republic of Moldova Statistical data Online available: http://www.statistica.md [Accessed: 01 August 2013]

[B2] Website of the World Bank World Bank database Online available: http://data.worldbank.org/ [Accessed: 01 August 2013]

[B3] Edwards N Improving the hospital system in the Republic of Moldova. Republic of Moldova Health Policy Series No.1 2011 Copenhagen: World Health Organization Regional Office for Europe

[B4] Jowett M Shishkin S Extending population coverage in the national health insurance scheme in the Republic of Moldova. Health Financing Policy Paper 2010/1 2010 Copenhagen: World Health Organization Regional Office for Europe

[B5] Turcanu G Domente S Buga M Richardson E Republic of Moldova: Health system review. Health Systems in Transition 2012 Copenhagen: World Health Organization Regional Office for Europe, Vol. 14 No.7 23211662

[B6] Sautenkova N Ferrario A Bolokhovets G Kanavos P Availability and affordability of medicines and assessment of quality systems for prescription of medicines in the Republic of Moldova. Republic of Moldova Health Policy Series No.6 2012 Copenhagen: World Health Organization Regional Office for Europe

[B7] Boerma WGW Snoeijs S Wiegers TA Baltag V Evaluation of the structure and provision of primary care in the Republic of Moldova. A survey-based project. Republic of Moldova Health Policy Series No.5 2012 Copenhagen: World Health Organization Regional Office for Europe

[B8] World Health Organization Equitable access to essential medicines: A framework for collective action. WHO Policy Perspectives on Medicines 2004 Geneva: World Health Organization

[B9] Bardelay D An ISDB survey to assess the degree of transparency of drug regulatory agencies Int J Risk Saf Med 1996 9 151 155 23510911 10.3233/JRS-1996-9304

[B10] Ratanawijitrasin S Wondemagegnehu E Effective drug regulation: a multicountry study 2002 Geneva: World Health Organization

[B11] Kaplan W Laing R Paying for pharmaceutical registration in developing countries Health Policy Plan 2003 18 3 237 248 10.1093/heapol/czg030 12917265

[B12] Olsson S Pal SN Stergachis A Couper M Pharmacovigilance activities in 55 low- and middle-income countries: a questionnaire-based analysis Drug Saf 2010 33 8 689 703 10.2165/11536390-000000000-00000 20635827

[B13] World Health Organization, Joint NLN-WHO Workshop Pharmaceuticals and the internet: Drug regulatory authorities’ perspective. NLN publication no.6 2002 Geneva: World Health Organization

[B14] Cornips C Rägo L Azatyan S Laing R Medicines regulatory authority websites: review of progress made since 2001 Int J Risk Saf Med 2010 22 2 77 88

[B15] World Health Organization WHO data collection tool for the review of drug regulatory systems. Regulatory support series no.11 2007 Geneva: World Health Organization

[B16] World Health Organization Practical Guidance for Conducting a Review. Regulatory support series no.12 2007 Geneva: World Health Organization

[B17] Ministerul Sanatatii al Republicii Moldova Ordin cu privire la reglementarea autorizarii produselor medicamentoase de uz uman si introducerea modificarilor postautorizare Monitorul Oficial Nr. 254–262, art Nr: 1555, Chisinau; 2012

[B18] Repulica Moldova Ministerul Sanatatii Ordin cu privire la aprobarea Regulilor de bună practică de fabricaţie a medicamentelor (GMP) de uz uman Monitorul Oficial Nr. 75–81, art Nr: 399, Chisinau; 2013

[B19] Website of the International Conference on Harmonisation of Technical Requirements for Registration of Pharmaceutical Products for Human Use The common technical document Online available: http://www.ich.org/products/ctd.html [Accessed 10 August 2013]

[B20] Management Sciences for Health Chapter 20: Quantifying pharmaceutical requirements MDS-3: Managing Access to Medicines and Health Technologies 2012 Arlington, VA: Management Sciences for Health

[B21] Ministerul Sanatatii al Republicii Moldova, Agentia Medicamentului si Dispozitiverol Medicale Resursele si activitatea sistemului farmaceutic al Republicii Moldova. Anuar statistic 2013 Chisinau: Agentia Medicamentului si Dispozitiverol Medicale

[B22] Website of the World Health Organization Regional Office for Europe Republic of Moldova: Areas of work Online available: http://www.euro.who.int/en/countries/republic-of-moldova/areas-of-work [Accessed 02 September 2013]

[B23] World Health Organization Effective drug regulation: What can countries do? 1999 Geneva: World Health Organization

